# Community Pop‐Up Clinic: Cascade of Care and HCV Treatment of Vancouver's Inner‐City PWID Populations

**DOI:** 10.1111/jvh.14023

**Published:** 2024-10-19

**Authors:** Shana Yi, Christina Wiesmann, David Truong, Shawn Sharma, Brian Conway

**Affiliations:** ^1^ Vancouver Infectious Diseases Center Vancouver British Columbia Canada; ^2^ Faculty of Health Sciences Simon Fraser University Burnaby British Columbia Canada

**Keywords:** community engagement, DAA, hepatitis C, multidisciplinary, people who inject drugs

## Abstract

Elimination of HCV infection as a public health concern by the end of this decade will require a concerted effort in all target populations, including drug‐users in the inner‐city. Several strategies have been proposed to identify, engage and provide HCV‐infected residents with antiviral therapy and maximise treatment and cure achievement. This study aims to assess the effectiveness of a multidisciplinary approach in delivering HCV treatment to people who inject drugs (PWID) within Vancouver's inner city. We have evaluated a novel approach, the Community Pop‐Up Clinic, for its ability to promote access to care and uptake of HCV therapy, with additional analyses of HCV reinfection and opioid‐related mortality. From January 2021 to August 2023, we evaluated 1968 individuals. 620 (31.5%) were found to carry HCV antibodies and of these, 474 (76.5%) were found to be viremic. Treatment engagement has been secured in 387 (81.6%). 326 (84.2%) have started treatment, 60 in the pre‐treatment phase and 1 died of an overdose in pre‐treatment. Of 326, 302 completed treatments, 18 are currently on treatment and 1 died of an overdose. Of 302 who completed treatment, 286 confirmed as cured (SVR 12), 16 are awaiting SVR 4, 2 had documented virologic relapse and 1 was reinfected. Three patients withdrew from treatment. By mITT, the cure rate is 286/288 (99.3%). We documented 2 overdose deaths over 326 PY. The data presented validates multidisciplinary programs such as ours aimed at treating HCV in inner‐cities and highlights societal benefits that could be achieved including lower overdose death rates.

## 1 Introduction

Hepatitis C Virus (HCV) stands as a global pandemic, impacting over 58 million people and is linked with significant morbidity and mortality. This viral infection can lead to severe liver diseases, including cirrhosis and liver cancer, contributing to a substantial public health burden [1] The World Health Organization (WHO) has set an ambitious goal to eliminate viral hepatitis, including HCV infection, as a public health concern by the end of this decade. To achieve this, WHO aims for 90% of infected individuals to be diagnosed and 80% to be successfully treated. Meeting these targets would significantly reduce incident infections by 90% and decrease disease‐associated mortality by 65% [1].

One of the major populations affected by HCV is people who inject drugs (PWID). It is estimated that over 15.6 million people inject drugs worldwide, with 6.1 million of them living with HCV infection [2]. This group faces multiple barriers to accessing healthcare, including stigma, criminalization, and lack of targeted healthcare services. Despite the advances in HCV therapy, particularly the development of highly effective direct‐acting antivirals (DAAs), many PWID remain untreated. The absence of proper structures to link diagnosed individuals to care, such as integrated health services that combine addiction treatment with HCV care, continues to impede the effective provision of antiviral treatment. Additionally, harm reduction strategies, such as needle exchange programs and opioid substitution therapy, are crucial in preventing new infections and need to be widely implemented and accessible [3].

Homelessness poses a significant challenge in North America, demanding special attention. In British Columbia alone, 26,240 individuals experienced homelessness at some point in 2021 [4]. Public health emergencies like COVID‐19 have exacerbated the situation, leading to a surge in the number of homeless individuals. The 2023 Greater Vancouver Homeless count found 4821 people self‐identified as homeless, a notable increase from 3634 individuals in 2020, marking a 32% rise over 3 years [5]. Homeless individuals confront elevated rates of health conditions and face heightened risks of all‐cause mortality due to the multiple daily challenges they face including financial challenges, mental health issues, and potential substance abuse disorders [6, 7]. Despite these pressing needs, this marginalised population continues to encounter barriers to accessing healthcare and often remains disconnected from care systems.

We have evolved our model of care to be more community‐based, with community pop‐up clinics (CPCs) conducted on a weekly basis. These clinics are held at various central locations in the inner city. At each clinic up to 30 individuals are seen and offered point‐of‐care diagnostic testing for HCV infection. Those who are found to be infected are offered immediate engagement in care to address medical, mental health, social and addiction‐related needs (including education about safer practices, naloxone training and access to agonist therapy and safe supply). This is supported by a physical clinic, Vancouver Urban Health Centre (VUHC) directly located in Vancouver's inner city. HCV treatment is delivered and monitored in this context.

Our study rests on two primary hypotheses. First, we propose that community‐based partnerships and targeted multidisciplinary care interventions will facilitate the engagement of vulnerable populations who are currently disengaged from healthcare, thereby enabling them to access curative HCV treatment. Second, we propose that these same partnerships will lead to engagement of a substantial number of individuals who are disconnected from addiction care, allowing them to benefit more fully from the measures already in place to address the opioid crisis and reduce the morbidity and mortality associated with street drug use.

## 2 Methods

### 2.1 Study Design

#### 2.1.1 Participants

Participants were 19 years or older, documented to be viremic with any HCV genotype at the time of enrollment, previously untreated for their infection and actively using or injecting drugs or having been documented to be doing so in the previous 3 months. Individuals with cirrhosis (as documented by a FibroScan measure > 12.5 kPa) or decompensated liver disease, pregnant or breast‐feeding women, those with a positive test for hepatitis B surface antigen (HBsAg) at the time of presentation, those judged to be too unstable to engage in care, unable to provide informed consent, or with a medical contraindication to the prescription of S/V were excluded. Women of child‐bearing potential were eligible to participate if sexually abstinent or using two effective methods of birth control. All participants provided informed consent before any study procedures were performed. The study protocol was approved by Advarra and was in accordance with the Declaration of Helsinki and International Conference on Harmonisation Good Clinical practice guidelines.

### 2.2 Participants Recruitment Through CPCs

Subjects were identified between March 2019 and August 2022 through weekly events referred to as CPCs in the inner city of Vancouver, Canada. These CPCs, held in various inner‐city locations such as shelters, community service centres, modular housing facilities, and single‐room occupancy dwellings within the Downtown East Side (DTES) of Vancouver, evaluate up to 30 adults over a 3‐h period. Eligible participants provide informed consent, sign a medical information release, and complete a demographic questionnaire. Testing is done using the OraQuick HCV Rapid Antibody Test, a single use fingerstick kit with a sensitivity and specificity exceeding 97%. Participants receive a $10 CDN gift card upon completing the test, irrespective of whether they choose to consult with the on‐site physician. Recruitment was briefly halted from March to June 2020 due to COVID‐19‐related healthcare restrictions. Otherwise, recruitment proceeded steadily without significant interruptions.

**FIGURE 1:**
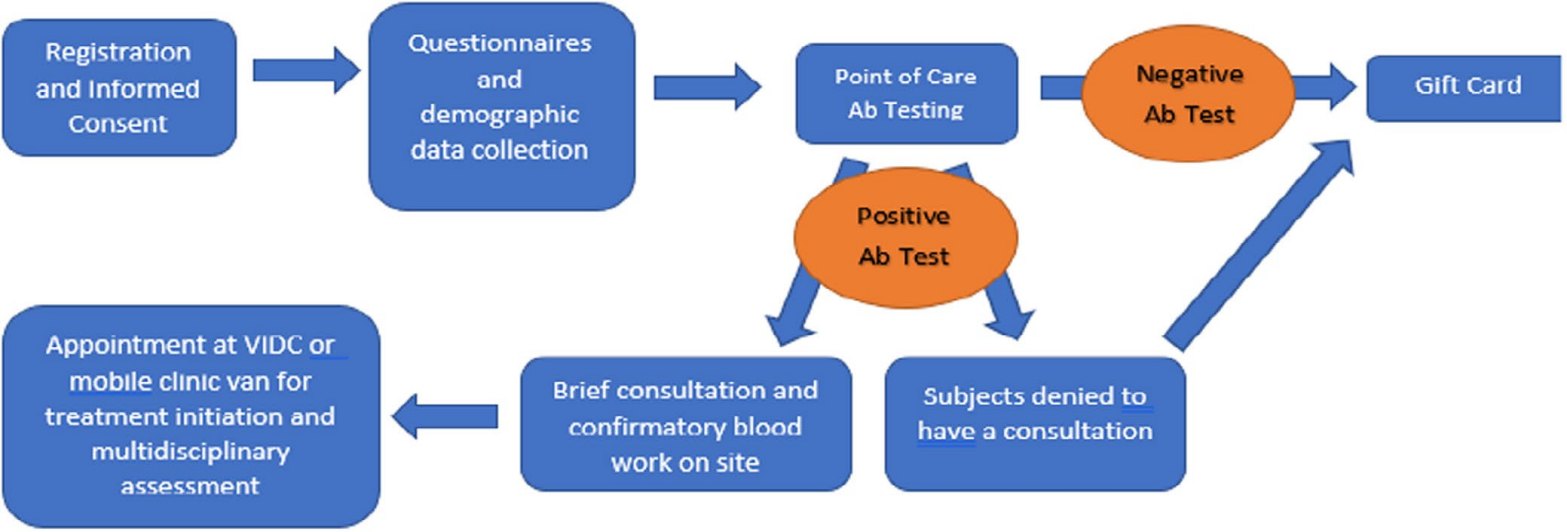
CPC organisational chart.

Participants with positive test results undergo confidential consultations. Prior to the consultation, their information is reviewed in provincial laboratory databases, subject to participant consent, to identify any history of hepatitis C (HCV) diagnosis and/or therapy in British Columbia (Figure 1). A comprehensive individual care plan is developed during the consultation, and participants have the option to schedule an appointment with a physician at Vancouver Infectious Diseases Centre (VIDC) within the next week, along with receiving a $10 CDN meal voucher. VIDC appointments encompass a variety of services, such as assistance with housing paperwork, government disability funding and nutritional support. The dedicated staff at VIDC address medical, psychological/psychiatric and addiction‐related needs. Non‐prescription medications, snacks, beverages, and protein drinks are provided free of charge. Baseline evaluations include confirming HCV RNA and genotype, standard laboratory testing, FibroScan transient elastography and any other clinically indicated assessments.

Free‐of‐charge DAAs are supplied through fully funded government programs, complemented by patient support initiatives. The treatments are overseen by Specialty Rx, a central pharmacy partner that offers flexible dispensing schedules to enhance adherence. Participants may be evaluated at VIDC during treatment for acute medical conditions, side effects, or adherence issues. Additional clinical and laboratory evaluations may be conducted as part of routine medical care. Following treatment completion, individuals continue to be part of the multidisciplinary care program to monitor the outcomes of HCV therapy.

### 2.3 Housing and Pharmacy Partnerships

VIDC has been active in inner‐city medicine for over 20 years. Vancouver's inner city is home to one of Canada's largest urban populations of First Nations people, with over 20% of our patients being Indigenous. We actively seek to serve this population and build trust within Indigenous communities. As part of our community partnership building, VIDC has established partnerships with the Lu'ma First Nation housing society and Atira women's housing to conduct CPCs in housing societies dedicated to Indigenous people and females, ensuring accessibility of care for the urban Indigenous and women's communities. Additionally, our healthcare providers who conduct weekly CPCs within buildings identified by their managers to house Indigenous individuals, including Lu'ma housing society, are fully trained to provide culturally safe healthcare, having completed San'yas Indigenous Cultural Safety Training. We also have a Memorandum of Understanding between Atira housing and VIDC to have a formal partnership through which to execute our programs with Atira housing sites spread across the inner‐city.

During each Community Pop‐Up Clinic event, our staff acknowledges and respects participants' Indigenous status and background. Our healthcare providers are fully aware of Indigenous‐specific and Indigenous‐led resources and are committed to connecting Indigenous patients with Indigenous‐focused resources, acting as a liaison if needed to facilitate this connection. VIDC is fully committed to providing patient‐centred and culturally safe care to marginalised populations who are disengaged from care. Through this, we offer opportunities for patients to discuss implementing cultural practices into their care plans to feel more engaged.

We have established a collaborative partnership with Lookout Housing Society and have formalised a service agreement appointing a case manager nurse for our CPC program and outreach initiatives. Presently, Lookout Housing Society oversees operations spanning 69 facilities, 50 housing programs, and 16 emergency shelters. Its service footprint extends to thousands of individuals residing within the Lower Mainland, encompassing 19 municipalities across Vancouver, Fraser Valley, and Vancouver Island, facilitated through a network of 144 programs. This partnership lays the groundwork for the deployment of a dedicated nurse at Lookout Housing Society sites visited during our CPC program, facilitating improved follow‐up and sustained patient engagement among those recruited from this institution. The pivotal role assumed by the case manager nurse entails orchestrating coordination and follow‐up efforts for our program initiatives focused on Lookout Housing Society‐monitored properties, thereby optimising patient care provisions.

The partnership established between the VIDC and SRX pharmacy marks a significant advancement in providing comprehensive care to our patients. With our satellite clinic, UHC, strategically located directly above SRX's Pier Pharmacy, we've created a seamless healthcare experience for our community. This proximity allows our patients unparalleled convenience, enabling them to receive assessments and promptly pick up their medications in one visit. Moreover, this collaboration extends beyond mere convenience. Pier Pharmacy plays a vital role in our patients' ongoing care by offering daily medication deliveries and ensuring adherence to treatment plans. Through close coordination with our physicians, the pharmacy ensures that patients receive timely reminders and support to pick up their medications, enhancing treatment compliance and ultimately improving health outcomes. This integrated approach exemplifies our shared commitment to multidisciplinary care and underscores the transformative potential of our partnership.

### 2.4 Outcomes

The primary efficacy endpoint of the study was the proportion of participants achieving SVR12, defined as HCV RNA below the limit of quantitation 12 or more weeks after completing treatments. This analysis was performed excluding participants who did not achieve SVR12 for reasons unrelated to their HCV infection or its treatment (modified intent‐to‐treat analysis, or mITT). Secondary endpoints of interest included rate of premature treatment discontinuation, loss to follow up during the study, mortality, HCV relapse and treatment failure and early HCV reinfection following demonstration of cure.

## 3 Statistical Analysis

For this retrospective evaluation of our CPC model, descriptive statistics were utilised to report on the primary and secondary outcomes.

## 4 Results

**FIGURE 2:**
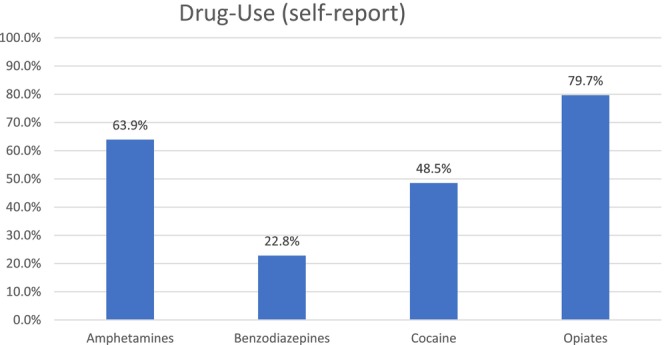
Drug type usage distributions (self‐reported).

From January 2021 to August 2023 (32 months), we conducted 112 CPCs and evaluated 1968 individuals. Demographic information was collected from 1388 individuals who voluntarily completed our questionnaire. We note median age 45 (20–93) years, 34.9% female, 32.3% indigenous (a key target population for intervention) and 50% Caucasian (Table 1). Almost 2/3 experience housing insecurity, with half having experienced a recent medically significant overdose. The majority have high school or greater education and over 60% have experienced incarceration. Over 80% smoke, while living in buildings that are, by city by‐law, non‐smoking. The drug use distribution was self reported with opiates being the majority of drug of choice at 79.7%, followed by amphetamines at 63.9%, cocaine and benzodiazepines at 48.5% and 22.8%, respectively (Figure 2). Almost all practise poly‐substance use.

**TABLE 1:** Demographic characteristics of CPC cohort.

Demographic	*N* = 1388
Age year (range)	45 (20–93)
Female	484 (34.9%)
Ethnicity	
Asian	71 (5.1%)
Black	48 (3.5%)
Indigenous	449 (32.3%)
White	696 (50.1%)
Other	124 (8.9%)
Unstable housing	869 (62.6%)
Education level	
Less than elementary	48 (3.5%)
Elementary	185 (13.3%)
High school	749 (54%)
College/University	406 (29.3%)
Incarceration history	818 (58.9%)
History of drug overdose	654 (47.1%)

**FIGURE 3:**
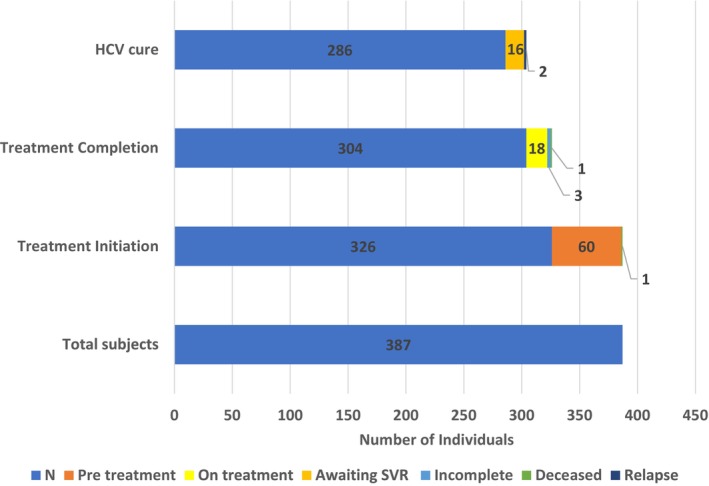
CPC cascade of care.

Of all participants, 620 (31.5%) were found to carry HCV antibodies. Of these, 474 (76.5%) were found to be viremic. Engagement in care has been secured in 387 cases (81.6%): 326 (84.2%) individuals have started treatment, 60 are in the pre‐treatment phase, and 1 had died of an overdose in the pre‐treatment phase (Figure 3). None have been lost to follow‐up. The median time from CPC attendance to HCV treatment initiation was 6 weeks. Of 326, 304 have completed the treatment, 18 remain on treatment, 3 stopped prematurely due to side effects and 1 died of an overdose during treatment. Of 304 subjects who have completed treatment, 286 are confirmed as cured (SVR 12), 16 are awaiting SVR status, 2 failed therapy with a documented virologic relapse (1 being cirrhotic) and 1 was cured and reinfected, a rate of 0.31/100 person‐years. By mITT analysis, the SVR rate is 99.3% (268/288). Overall, in this vulnerable population with 6–7 opioid overdose deaths/day, we only documented 2 overdose deaths over 326 PY of overall follow‐up.

## 5 Discussion

Many different models with which to increase engagement and adherence of PWID in HCV DAA treatment have been used around the world. In the United States, one study investigated the use of patient navigation vs. mDOT achieved mITT rates of 74% and 76%, respectively, and ITT rates of 60% and 62%, respectively [8]. In Tayside Scotland, a multi‐faceted approach involving community engagement specifically aiming at treating PWID for HCV. Using this approach their ITT rate was 80.9% and their mITT rate was 91.4% [9]. To date, through our community‐centred approach we have achieved an ITT rate of 81.6% with an mITT rate of 99.3%. These results demonstrate that a community‐centred approach is required to engage high numbers of PWUD in DAA treatment. Further, our unique partnerships with a community pharmacy which can offer flexible dispensing schedules to enhance adherence, and the close proximity of our UHC satellite clinic to where our most vulnerable patients are living, have helped us to achieve unprecedented levels of treatment adherence and SVR in our study population.

An integral component of our CPC approach to diagnosis and care is engagement of patients undergoing DAA treatment in addition therapy, including access to opiate agonist therapy (OAT) and hydromorphone as a “safer supply” opiate. Previous work found that OAT adherence was a major predictor of HCV DAA initiation [10, 11] and, in turn, that DAA treatment initiation was associated with longer duration of OAT [12]. This suggests that the prescribing of OAT may, in part, contribute to the high rates of treatment adherence achieved in our study. It is also notable that we see low rates of overdose deaths in our study population (2 overdose deaths over 326 PY of overall follow‐up), a population in which there is an average of 6–7 opioid overdose deaths/day. This is consistent with previous findings that individuals on OAT therapy have lower rates of overdose [13, 14].

While this study presents a successful community intervention for HCV treatment among PWID in Vancouver's inner city, several factors must be considered when extrapolating these results to other regions or countries. The population dynamics, healthcare infrastructure and accessibility of harm reduction services, such as opioid agonist therapy and safe supply programs, vary significantly across regions [15]. Therefore, replicating these outcomes may depend on the local healthcare system's ability to integrate similar multidisciplinary care models and engage marginalised populations. Nonetheless, the underlying principles of community outreach, multidisciplinary care and partnerships with local housing and pharmacy services offer a scalable approach, provided sufficient resources and political will are available in other areas.

Regarding HCV elimination, the WHO's goal of eliminating HCV as a public health threat by 2030 requires innovative strategies to engage populations such as PWID, who are often disconnected from healthcare services [16, 17, 18]. The high engagement and cure rates achieved in our study demonstrate that community‐centred models, like CPCs, can contribute significantly to reaching elimination targets by reducing the number of undiagnosed and untreated cases. Our intervention not only treats active infections but also facilitates access to harm reduction services, likely reducing reinfection and transmission rates within this high‐risk population, aligning with the broader global effort to lower incidence and mortality.

Finally, assessing cost‐effectiveness is crucial for wider adoption of this intervention. While we do not include a formal cost analysis, other similar models of care for engaging PWIDs in HCV treatment have been modelled to be cost effective [19, 20, 21]. Because the CPC model leverages existing community partnerships, this likely lowers operational costs compared to more traditional hospital‐based treatment settings. The use of free‐of‐charge DAAs through government‐funded programs also reduces treatment expenses, and as previous analyses have shown. Further research should include a detailed economic evaluation to provide a more concrete understanding of the financial viability of scaling such interventions. Estimating the cost per patient cured, as well as the broader societal benefits of reduced HCV transmission and improved overall health, would provide valuable insights for policymakers considering similar interventions elsewhere.

## 6 Limitations

Firstly, the reporting and collection of patient‐level data in self‐reported drug use, history of overdose and alcohol use are subject to considerable bias arising from incomplete information or stigma associated with substance use. Regardless, studies have suggested that collection of substance use data and other stigmatised behaviours yield reliable results. Secondly, one could also argue the fact that our HCV treatment program and recruitment strategies are excessive and would not be able to be reproduced due to financial and human resource limitations. However, it could be argued that the basic clinical infrastructure that we provide should be the standard for health care delivery to inner city populations.

## 7 Conclusion

In the context of an established, multidisciplinary clinic serving the inner city, a 4‐h/week initiative was conducted to identify HCV‐infected individuals and engage them in care. By any measure, this constitutes a highly vulnerable population, based on addiction‐related and social measures. The CPC events are clearly well targeted with over 30% of participants being infected with HCV. Our strategy for engagement in care after demonstration of viremia is highly successful, approaching a rate of 90%, with a high rate of initiation of therapy in the short term, and retention in care once treatment is initiated. A high cure rate is achieved (99%), comparable to that reported in clinical trials and in real world cohorts among less challenging patient populations. Reinfection rates, at least in the short term, are extremely low, possibly related to maintenance of engagement in care leading to less risky drug use behaviours, which may also reduce the risk of opiate overdose mortality. We propose three key parameters for the evaluation of the performance of outreach programs among vulnerable populations: prevalence of HCV infection in the target population; number of individuals receiving HCV treatment per conducted event; and success/cure rate of HCV therapy. Programs such as ours will play an important role in HCV elimination as well as in contributing to the increased stability of an inner‐city population with many urgent needs that transcend HCV infection.

## Author Contributions

**Shana Yi:** writing, visualisation, reviewing, operations and editing. **Christina Wiesmann:** writing, reviewing, and editing. **David Truong:** operations, reviewing and editing. **Shawn Sharma:** operations. **Brian Conway:** conceptualization, writing, operations, reviewing and editing.

## Consent

All cohort participants provided written informed consent to the access of their personal health numbers and the questionnaire survey.

## Conflicts of Interest

Dr. Conway has received research grants, honoraria and/or acted as a remunerated advisor for AbbVie, Astra Zeneca, Gilead Sciences, GSK, Indivior Canada, Merck, Moderna, Sanofi Pasteur, Seqirus and ViiV Healthcare. In particular, AbbVie and Gilead Sciences have funded the community pop‐up clinic program in a direct way. D.T. has received honoraria and acted as a renumerated advisor for AbbVie and Gilead Sciences. S.Y., C.W. and S.S. have declare no conflicts of interest.

## Data Availability

De‐identified original data is available through the corresponding author.
